# Daily versus as-needed inhaled corticosteroid for mild persistent asthma (The Helsinki early intervention childhood asthma study)

**DOI:** 10.1136/adc.2007.116632

**Published:** 2007-07-18

**Authors:** M Turpeinen, K Nikander, A S Pelkonen, P Syvänen, R Sorva, H Raitio, P Malmberg, K Juntunen-Backman, T Haahtela

**Affiliations:** 1 Department of Allergy, Helsinki University Hospital, Finland; 2 AstraZeneca R&D, Lund, Sweden

## Abstract

**Objective:**

To compare the effect of inhaled budesonide given daily or as-needed on mild persistent childhood asthma.

**Patients, design and interventions:**

176 children aged 5–10 years with newly detected asthma were randomly assigned to three treatment groups: (1) continuous budesonide (400 μg twice daily for 1 month, 200 μg twice daily for months 2–6, 100 μg twice daily for months 7–18); (2) budesonide, identical treatment to group 1 during months 1–6, then budesonide for exacerbations as needed for months 7–18; and (3) disodium cromoglycate (DSCG) 10 mg three times daily for months 1–18. Exacerbations were treated with budesonide 400 μg twice daily for 2 weeks.

**Main outcome measures:**

Lung function, the number of exacerbations and growth.

**Results:**

Compared with DSCG the initial regular budesonide treatment resulted in a significantly improved lung function, fewer exacerbations and a small but significant decline in growth velocity. After 18 months, however, the lung function improvements did not differ between the groups. During months 7–18, patients receiving continuous budesonide treatment had significantly fewer exacerbations (mean 0.97), compared with 1.69 in group 2 and 1.58 in group 3. The number of asthma-free days did not differ between regular and intermittent budesonide treatment. Growth velocity was normalised during continuous low-dose budesonide and budesonide therapy given as needed. The latter was associated with catch-up growth.

**Conclusions:**

Regular use of budesonide afforded better asthma control but had a more systemic effect than did use of budesonide as needed. The dose of ICS could be reduced as soon as asthma is controlled. Some children do not seem to need continuous ICS treatment.

Most children with asthma experience their first symptoms before 7 years of age.^1^ Studies of adults and children with asthma have shown that some functional reversibility may be lost if anti-inflammatory treatment is postponed.^2^^–^4 The anti-asthmatic effect of inhaled corticosteroids (ICS) has been demonstrated in long-term intervention studies,^5^^–^10 and these findings have led to ICS becoming the mainstay of treatment for persistent asthma.^11^ ^12^ However, high-dose ICS may have systemic effects such as reduction in height velocity^6^^–^8 13 and adrenal insufficiency.^14^

In an 18-month intervention, we compared two budesonide therapeutic regimens with a control group treated with a fixed dose of disodium cromoglycate (DSCG). The study was designed to evaluate the anti-asthmatic efficacy and systemic effect of daily versus as-needed budesonide in the treatment of early, mild persistent asthma in children.

## METHODS

Children between 5 and 10 years, all Caucasians, were included, if they presented symptoms such as wheezing, prolonged cough or shortness of breath, suggesting asthma for at least 1 month before entry into the study, and significant bronchial reversibility. The latter was defined as at least a 20% diurnal variation in repeatable peak expiratory flow (PEF) measurements, or at least a 15% increase in PEF at least three times within 2 weeks of home recording, or at least a 15% increase in forced expiratory volume in 1 second (FEV_1_) 15 min after inhalation of a β_2_-agonist, or at least a 15% decline in FEV1 in an outdoor exercise test in the clinic.^15^ According to the symptoms and lung-function tests, the majority of children could be categorised as having mild persistent asthma.^16^ Children with acute asthma, an FEV_1_<50%^17^ and with treatment during the preceding 2 months with ICS, cromones, leukotriene modifiers or long-acting β_2_-agonists were excluded. The total cumulative doses of previously used ICS must not have exceeded 36 mg, 12 mg of nasal corticosteroids or oral doses equivalent to 200 mg prednisolone.

**Figure 1:**
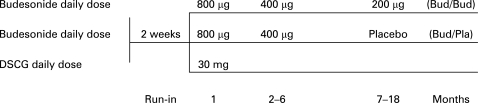
Study design. The daily dose of budesonide was divided into two doses, DSCG into three doses.

The 18-month study was of a controlled, randomised, double-blind, parallel-group, single-centre design including a 2-week run-in period. Two blinded treatment regimens were initiated with inhaled budesonide via a dry-powder inhaler (Pulmicort Turbuhaler, AstraZeneca, Lund, Sweden), and one open-label treatment regimen was initiated with DSCG via a pressurized metered dose inhaler (pMDI; Lomudal with Fisonair spacer, Aventis Pharma, Holmes Chapel, UK). Patients were randomised as to treatment in balanced blocks as generated by a computer program. During the 2-week run-in period, all enrolled patients received a short-acting β_2_-agonist, terbutaline (Bricanyl Turbuhaler, 0.25 mg/dose, AstraZeneca, Lund, Sweden) as needed. After the run-in period, children were assigned to one of three treatment groups: (1) continuous budesonide group, receiving budesonide (400 μg twice daily for the first month, then 200 μg twice daily for 5 months) followed by low-dose budesonide (100 μg twice daily for 12 months); (2) budesonide/placebo group, where patients received identical budesonide treatment as Group 1 for the first 6 months followed by placebo for 12 months; and (3) DSCG control group, where patients received DSCG 10 mg three-times daily for 18 months (fig 1).

All patients were given rescue medication of terbutaline 0.25 mg per dose as needed. For all groups, during exacerbations of asthma, study medication was replaced by budesonide 400 μg twice daily for 2 weeks. Children were withdrawn from the study and given individually tailored therapy if treatment of their exacerbations remained insufficient.

The study was performed in accordance with the Declaration of Helsinki, and was approved by the local ethics committee. Written, informed consent was obtained from each patient’s parent(s) or legal guardian and from the patient.

For the budesonide treatment groups, treatment compliance was recorded using a home spirometer (Vitalograph Data Storage spirometer, Vitalograph, Buckingham, UK), which recorded the peak inspiratory flow via Turbuhaler (PIF_TBH_) each time a dose of the drug was taken.^18^ In the DSCG group, the returned pMDI drug canisters were counted and weighed every 3 months.

The primary efficacy variable was morning PEF. Secondary efficacy variables were FEV_1_, the number of asthma exacerbations, asthma-free days and rescue medication use. Morning PEF was measured daily at home. FEV1 was measured at the clinic visit every third month. An asthma exacerbation was defined as an increase in symptoms that were not controlled with six doses of rescue terbutaline per 24 h that caused the parent to contact the clinic. All parents were provided with a 24-h emergency telephone number. At the clinic, patients were examined by a paediatrician, who decided whether an exacerbation had occurred and, if so, replaced the regular medication with a 2-week course of budesonide 400 μg twice daily. The treatment of an exacerbation was considered insufficient if the symptoms did not subside during the 2-weeks’ budesonide inhalations that caused the parent to contact the clinic. If an oral or parenteral corticosteroid was needed, the child received individual treatment and was withdrawn from the study.

All patients recorded their PEF rate daily, as measured by a home spirometer, before taking study medication. They also recorded their asthma symptoms using a visual analogue scale (0–10) and use of rescue medication. An asthma-free day was defined as a 24-h period without use of rescue medication and with a symptom score <2. Standard laboratory spirometry (Spirotrac III, Vitalograph) was performed at all clinic visits.^19^

The primary indicator of systemic effect was the standing-height velocity, which was measured at each clinic visit using a stadiometer (Holtain, Crymych, UK) following a standardised procedure. Children with Tanner stage I-II at baseline were included. Tanner stage of sexual development is scored from I (pre-adolescence) to V (adult characteristics).^20^ Standing height was compared with Finnish reference values.^21^ Other indicator was body mass index (kg/m^2^).

The sample size was determined by power calculations for morning PEF. A clinically significant change was assumed to be 40 l/min over an 18-month period. With 60 patients per treatment group, there was a 90% chance of detecting a difference of 24 l/min between treatments. The analysis of growth was performed on the complete study population (excluding pubertal children). All other variables were analysed using intention-to-treat principles, that is, all patients who had taken at least one dose of study medication and had data for the required period(s). Withdrawn patients were handled using last value extended, within period. Comparisons between the combined budesonide groups and the DSCG group were made for months 1–6; from 7–18 months comparisons were made between all three groups.

For most variables, treatment groups were compared using analysis of variance (ANOVA) with fixed factor treatment and baseline values as covariates. For growth variables, sex was included as an additional factor in the analysis. Time to first asthma exacerbation and time to withdrawal were compared using the log-rank test. The number of exacerbations was compared using a Poisson regression model with fixed factor treatment, time in study as an offset and adjustments made for overdispersion.

## RESULTS

**Figure 2:**
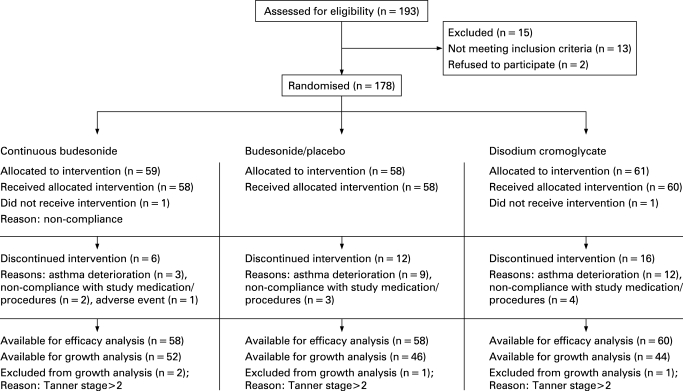
The flow of the participants through the trial.

A total of 176 children were enrolled in the study. There were no significant differences between treatment groups in any baseline measures (table 1). During the run-in period, the mean use of terbutaline was about one dose every 2 days in all treatment groups. Three patients were withdrawn because of asthma deterioration during continuous budesonide treatment after 6 months of the study. In the budesonide /placebo group, nine children were withdrawn because of asthma deterioration, all after 6 months of treatment. In the DSCG group, eight children were withdrawn during the first 6 months of the study, and four children thereafter (continuous budesonide versus DSCG; p = 0.026). One child on placebo and one child on DSCG were hospitalised, because of deterioration in their asthma. The numbers of patients withdrawn from the treatment groups for reasons not related to asthma were three in the continuous budesonide group, three in the budesonide/placebo group, and four in the DSCG group. The flow of the participants through the trial is presented in the fig 2. The mean treatment compliance for the three treatment groups decreased linearly from an initial level of ∼90% to a mean level of ∼60% towards the end of the study. This was matched by a subsequent reduction in the amount of drug used during the study. Children in the continuous budesonide and budesonide/placebo treatment groups achieved a mean PIF_TBH_ of 60 l/min during the study period.

**Table 1:** Baseline characteristics of treatment groups*

Treatment group	Continuous budesonide (n = 58)	Budesonide/placebo (n = 58)	Disodium cromoglycate (n = 60)
Age (years)	7.0 (5 to 10)	6.7 (5 to 10)	6.9 (5 to 10)
Male (%)	59	66	54
Tanner pubertal stage I/II	58/1	58/1	61/2
Standing height (cm)	128.4 (108 to157)	125.1 (106 to 148)	125.6 (105 to 148)
Standing height, standard deviation scores (SDS)	0.04 (−0.32 to 0.54)	0.03 (−0.30 to 0.39)	0.04 (−0.43 to 0.32)
Body mass index (kg/m^2^)	17.5	16.9	16.9
Skin prick test positive (n)	35	41	36
Duration of symptoms (months†)	12.8 (1.1 to 70.5)	11.3 (2.0 to 76.4)	11.7 (3.0 to 70.8)
Wheeze ever (n)	35	42	33
Asthma symptom score (0–10)‡	1.5 (0.0 to 5.5)	1.7 (0.0 to 4.5)	1.9 (0.0 to 5.7)
Rescue medication, dose/24 h‡	0.47 (0 to 4.0)	0.55 (0 to 3.7)	0.68 (0 to 2.8)
Morning PEF rate (l/min)‡	182 (78 to 301)	176 (68 to 313)	184 (94 to 363)
Morning PEF (% predicted value)‡	76 (43 to 105)	77 (42 to 112)	79 (54 to 107)
FEV_1_ (L†)	1.43(0.89 to 2.15)	1.32 (0.72 to 2.36)	1.37 (0.63 to 2.45)
FEV_1_ (%† predicted value)	87 (57 to 111)	82 (52 to 107)	83 (57 to 107)
FVC (% predicted value)	90 (64 to 112)	87 (57 to124)	89 (56 to 120)

*Values are means with range in parentheses, unless otherwise stated; †no correlation between duration of the symptoms and FEV_1_. ‡Data from the run-in period. FEV_1_, forced expiratory volume in 1 s; FVC, forced vital capacity; PEF, peak expiratory flow rate.

After 6 months, the morning PEF values (l/min) of the budesonide groups improved by 6.6% and by 6.1% in the DSCG group. After 18 months, the increase was 10.3% in the continuous, 10.0% in the budesonide/placebo and 12.5% in the DSCG group. No significant differences were observed between the groups at any time point. After 6 months of treatment, improvement in FEV_1_ in litres in the clinic was significantly greater in the budesonide groups than in the DSCG group (9.6 versus 5.9%; p = 0.012). From baseline to 18 months, FEV_1_ improved by 18.2%, in the continuous, by 16.9% in the budesonide/placebo and by 17.3% in the DSCG group without any significant differences.

Over the 18-month study period, 364 exacerbations of asthma were recorded in 133 patients. During the first 6 months of treatment, children receiving budesonide had significantly fewer exacerbations compared with children in the DSCG group (table 2). During months 7–18, the continuous budesonide group (ie, children on low-dose budesonide) had significantly fewer exacerbations than either the budesonide/placebo group (ie, children given the placebo) or the DSCG group (table 2).

**Table 2:** Number of exacerbation episodes

Treatment		No of patients analysed*		Exacerbations/patient** (95% CI)	p Value		
**Months 1–6**							
Budesonide		115		0.32 (0.22 to 0.46)			
DSCG		60		1.24 (0.95 to 1.63)	<0.001		
**Months 7–18**							
Bud/Bud	57		0.97 (0.70 to 1.34)				
Bud/placebo (Budesonide as needed)	58		1.69 (1.31 to 2.18)				
DSCG	51		1.58 (1.20 to 2.08)				
Bud/Bud vs Bud/placebo						0.008	
Bud/Bud vs DSCG						0.023	
Bud/placebo vs DSCG						0.728	

*The total effective number of patients analysed. **The mean number of exacerbations divided by the number of patients in the group. Bud, budesonide.

**Figure 3:**
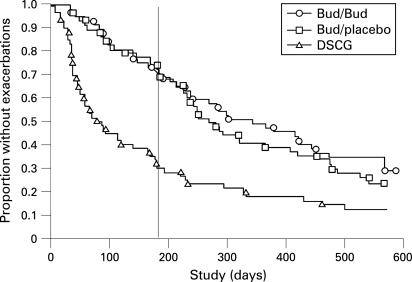
Kaplan–Meier plot of the time to first exacerbation for the continuous budesonide (O, n = 57), budesonide/placebo (□, n = 58) and disodium cromoglycate (Δ, n = 60) treatment groups during the 18-month study. The median time to the first exacerbation was significantly longer for both the continuous budesonide (344 days) and the budesonide/placebo (268 days) groups compared with the DSCG group (78 days) (p<0.001 for each). The vertical line indicates the time point (180 days) when budesonide treatment was changed to the low-dose regimen or to placebo. After 180 days, the median time to the next exacerbation was 233 days for the continuous budesonide group, 138 days for the budesonide/placebo group and 131 days for the DSCG group (continuous budesonide and DSCG; p = 0.03).

The median time to the first exacerbation was significantly longer for both the continuous budesonide (344 days) and the budesonide/placebo (268 days) groups compared with the DSCG group (78 days) (p<0.001 for each) (fig 3). After 180 days, the median time to the next exacerbation was 233 days for the continuous budesonide group, 138 days for the budesonide/placebo group (ie, during placebo) and 131 days for the DSCG group (continuous budesonide and DSCG; p = 0.03).

At 6 months, the mean number of asthma-free days increased more in budesonide group than in the DSCG group (table 3). During months 7–18, the mean number of symptom-free days increased significantly in the continuous budesonide group compared with the DSCG group.

**Table 3:** Asthma-free days after the run-in period

Treatment	No of patients analysed*	Mean change in asthma-free days, % ** (95% CI)			p Value		
**Months 1–6**							
Budesonide	114	+20.1 (+14.9 to +25.4)					
DSCG	60	+4.1 (−3.2 to +11.3)			0.001		
**Months 7–18**							
Bud/Bud	55		+29.2 (+21.2 to +37.2)				
Bud/placebo (Budesonide as needed)	58		+19.6 (+11.8 to +27.4)				
DSCG	51	+11.6 (+3.3 to +19.9)					
Bud/Bud vs Bud/placebo					0.092		
Bud/Bud vs DSCG					0.003		
Bud/Placebo vs DSCG					0.166		

*The total effective number of patients analysed; **Mean change in asthma-free days compared with the baseline.

During the first 6 months, compared with the run-in period, both budesonide groups used significantly less rescue terbutaline (−0.29 doses/day) than the DSCG group (−0.07 doses/day) (p = 0.012). During months 7–18, the decline was –0.29 doses/day in the continuous budesonide group, −0.22 doses/day in the budesonide/placebo group and −0.18 doses/day in the DSCG group, with no significant differences between the groups.

**Figure 4:**
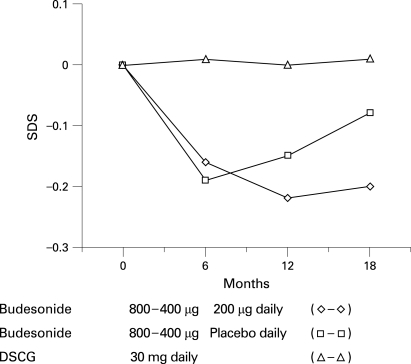
Mean change in standing height (SDS) over the 18-month study period for the continuous budesonide (◊, n = 50), budesonide/placebo (□, n = 45) and disodium cromoglycate (DSCG) (Δ, n = 43) treatment groups. 1–6 months, both budesonide groups versus DSCG, p<0.001; 7–18 months, continuous budesonide group versus budesonide/placebo group, p = 0.016. Note the fast height velocity during months 7–18 in the budesonide/placebo group.

From baseline to 6 months, the mean standing–height velocity in the budesonide groups was 2 cm/year slower than in the DSCG group (p<0.001). From 7 to 18 months, height velocity increased in both budesonide groups, with the mean height velocity being greater for the budesonide/placebo group (ie, during placebo) than the continuous budesonide group (6.2 versus 5.6 cm; p = 0.016). After 18 months of treatment, children receiving DSCG had grown, on average, 1.0 cm more than children in the continuous budesonide group (8.8 versus 7.8 cm; p = 0.008) and 0.6 cm more than children in the budesonide/placebo group (ie, during placebo) (8.8 versus 8.2 cm; p = 0.048). Development of standing height is presented as standard deviation scores (SDS) in fig 4. No significant differences in body mass index were observed between treatment groups at any time point.

## DISCUSSION

The artificial nature of the research protocol in our study, as in many other asthma studies with drug interventions, does not pay attention to the evolution of individual disease. Exclusion criteria used in this study affect the selection of children. During the study, the selection was further affected by the withdrawal criteria. Furthermore, a “true” placebo group is impossible to arrange because asthma exacerbations cannot be left untreated, and glucocorticoids used for the treatment of exacerbations might influence the individual evolution of asthma.

We consider that, clinically, the dominant phenotype of the children examined is mild persistent asthma according to the present guidelines. Some children with moderate persistent asthma were included, as consecutive patients fulfilling the inclusion criteria were allocated to the treatment groups. Within the treatment groups, every patient received fixed doses for a predetermined time despite the individual phenotype of asthma. However, in our study, the treatment regimen could be modified individually by 2-week courses of budesonide given as needed.

Cessation of inhaled budesonide maintenance treatment has previously been shown to result in a worsening of the disease and a decline in lung function in children with persistent moderate to severe asthma.^22^ In the present study of newly detected mild persistent asthma, a proportion of children had a low number of exacerbations during this intermittent treatment with budesonide. The exacerbation rate during months 7 to 18 in this budesonide/placebo group was similar to that of the DSCG group. In the present study, two weeks’ budesonide given when needed, after the initial regular treatment with budesonide, seems to produce an anti-exacerbation effect comparable with the continuous use of DSCG. However, most withdrawals in the DSCG were early, in contrast to late withdrawals in the regular budesonide and budesonide/placebo groups. This might select more mild phenotypes of asthma to the DSCG group for the last 12 months of treatment and artificially improve the results of DSCG compared with placebo or low-dose budesonide treatments.

While treatment of patients in the DSCG group was open, exacerbations were diagnosed and treated in the same way as in the other two treatment groups. Treatment in the DSCG group was not associated with measurable systemic effects. However, it was associated with the highest number of asthma exacerbations and withdrawals from the study. The initially high number of exacerbations suggests that DSCG is not suitable to start treatment of newly detected childhood asthma.

No significant differences between treatment groups were observed in the morning PEF values at any time point during the study. This suggests that morning PEF is not a very sensitive efficacy parameter in long-term studies in children with mild asthma as suggested previously.^7^ During the first 6 months of the study, FEV_1_ in litres improved significantly more in the budesonide groups than in the DSCG group. However, at the end of the study the differences in FEV_1_ disappeared despite significant differences in the number of exacerbations. This is in agreement with previous observations of changes in FEV_1_ in litres between the treatments with budesonide, nedocromil and placebo.^7^ The use of FEV1 values measured in litres has been recommended because predicted values depend on height, which may be affected by ICS.^7^

Our results confirm previous observations of a small initial decline in height velocity during treatment with ICS used at comparable doses, followed by normal height velocity.^7^ Decline in height velocity without catch-up growth has been recently observed even during regular low ICS dosage.^8^ However, another study suggests that children treated regularly with budesonide attain their predicted final adult height.^23^ In the present study, height velocity was dose-related; during the low-dose budesonide and placebo treatments, the systemic effect of the initial high-dose budesonide were reduced. In the present 18 months follow-up study, standing height velocity was normalised during low-dose budesonide treatment within 1 year of commencement of treatment. The height velocity increased, however, more rapidly during the placebo treatment than during the low-dose budesonide treatment, suggesting catch-up of the initial loss in standing height.

While long-term maintenance therapy with low-dose ICS is recommended for mild persistent asthma,^7^ ^8^ ^24^ ^25^ some children do not seem to need continuous inhaled corticosteroid treatment. Advantages of this treatment strategy include a reduced risk of ICS-related growth suppression. Intermittent courses of inhaled or oral corticosteroids have been suggested recently for adults with mild persistent asthma.^26^

Regular use of budesonide afforded better exacerbation control but more systemic effect than intermittent use of budesonide given as needed or regular DSCG treatment. No significant differences in the morning PEF and FEV_1_ in litres or in asthma-free days were observed between the regular or intermittent budesonide treatments during months 7–18. These findings suggest that the overall anti-asthmatic effect of the intermittent budesonide treatment might be intermediate between the regular low-dose ICS and DSCG treatments. The dose of ICS could be reduced as soon as asthma is controlled. Some children do not seem to need continuous ICS treatment.

**What is already known on this topic**

It is still debated whether mild asthma in adults needs regular treatment with inhaled corticosteroids.

**What this study adds**

Some children who achieve good initial control of their mild asthma does not seem to need continuous treatment with inhaled corticosteroids.
